# Potential impact of *NUCB2* genetic variants with the clinicopathological characteristics of prostate cancer

**DOI:** 10.7150/ijms.135299

**Published:** 2026-07-22

**Authors:** Yung-Shu Lee, Tien-Huang Lin, Chia-Yen Lin, Shun-Fa Yang, Chiao-Wen Lin, Chih-Hsin Tang

**Affiliations:** 1 Institute of Oral Sciences, Chung Shan Medical University, Taichung, Taiwan.; 2 Department of Urology, Taipei City Hospital Heping Branch, Taipei City, Taiwan.; 3 School of Post-Baccalaureate Chinese Medicine, Tzu Chi University, Hualien, Taiwan.; 4 Department of Urology, Buddhist Tzu Chi General Hospital Taichung Branch, Taichung, Taiwan.; 5 Division of Urology, Department of Surgery, Taichung Veterans General Hospital, Taichung, Taiwan.; 6 School of Medicine, Chung Shan Medical University, Taichung, Taiwan.; 7 Institute of Medicine, Chung Shan Medical University, Taichung, Taiwan.; 8 Department of Medical Research, Chung Shan Medical University Hospital, Taichung, Taiwan.; 9 Department of Dentistry, Chung Shan Medical University Hospital, Taichung, Taiwan.; 10 Department of Pharmacology, School of Medicine, China Medical University, Taichung, Taiwan.; 11 Department of Medical Laboratory Science and Biotechnology, Asia University, Taichung, Taiwan.; 12 Chinese Medicine Research Center, China Medical University, Taichung, Taiwan.

**Keywords:** nesfatin-1, *NUCB2*, prostate cancer, genetic polymorphisms

## Abstract

The most prevalent illness in men is prostate cancer, which risk increases with age and obesity. The precursor *NUCB2* gene produces the adipokine nesfatin-1, which was first found in hypothalamic neurons. It is currently unclear how *NUCB2* polymorphisms, cancer-promoting lifestyle factors, and prostate cancer are related. We investigated the relationship between clinicopathological features and 4 *NUCB2* gene polymorphisms in prostate cancer when compared to healthy individuals. Compared with the wild-type T/T genotype, carriage of at least one G allele (T/G or G/G genotypes) at the *NUCB2* SNP rs10766383 was linked with a declined risk of clinical T3+T4 stage, pathologic T3+T4 stage, and perineural invasion. In addition, the TG/GG genotypes at rs10766383 were also associated to a lower risk of clinical T3+T4 stage and perineural invasion in patients with biochemical recurrence. Importantly, GTEx data indicated that the wild-type TT homozygous genotype was linked with markedly higher *NUCB2* levels compared to the GG allele of variant rs10766383 variant in mucosa and whole blood tissues. Thus, the *NUCB2* SNP rs10766383 may play a protective function against prostate cancer progression.

## Introduction

Prostate cancer is the most typical disease in males, and its risk elevates with age and obesity 1-3. By 2050, there will likely be 300,000 new prostate cancer patients in the United States. More than half of prostate cancer patients ultimately develop bone metastases, notably in advanced stages, even though many first show up with organ-confined disease 2, 4-6. In addition, bone, lymph nodes, and distant organs are common sites of metastasis, which results in a poor prognosis and decreased survival. Many individuals with metastatic cancer develop treatment-resistant disease, despite the five-year associate survival rate for combined diagnosed cases of prostate cancer is 98%. Prostate cancer metastases are a critical cause of mortality and can markedly reduce patients' quality of life 7-9. The development of early diagnostic and prevention strategies would be aided by a thorough understanding of the mechanisms behind distant metastasis and malignant progression in prostate cancer 10, 11.

There is mounting evidence that obesity and cancer are related 12, 13. The malignancies where this relationship is most apparent are pancreatic, colorectal and prostate cancers 12, 14, 15. Additionally, obesity can alter the cancer's microenvironment, accelerating its growth 16. Adipokines, bioactive compounds secreted by adipose tissue, regulate numerous pathological processes 17. Among other characteristics of cancer, adipokines have an impact on apoptosis, angiogenesis, differentiation, invasion, proliferation and metastasis 18-20. Originating from its precursor *NUCB2*, nesfatin-1 is an 82-amino acid polypeptide that was initially identified in hypothalamus neurons 21. Other peripheral tissues that express nesfatin-1 include adipose tissue, the ovaries, the duodenum, and the pancreas 22. Nesfatin-1 has been proposed as an anorexigenic peptide having antihyperglycemic, antioxidant and anti-inflammatory characteristics 23, 24. Increased expression of *NUCB2* has been seen in colon, endometrial, prostate, thyroid and breast cancers, according to recent research 25. These results show that nesfatin-1 levels are strongly associated with a poor prognosis, as shown by the development of metastases and a lower life expectancy free from recurrence.

A single nucleotide variant that appears at a particular site in the genome is known as a single nucleotide polymorphism (SNP) 26. SNP distribution frequency analysis between patient cohorts is commonly used to predict the risk and prognosis of diseases like cancer 27, 28. There is no information on the connections between prostate cancer, *NUCB2* gene polymorphisms, and carcinogenic behavioral factors. Thus, this investigation examined the functions of *NUCB2* gene polymorphisms and carcinogenic lifestyle variables on the risk of progressing prostate cancer in a Taiwanese population.

## Materials and Methods

### Study participants

This study examined blood samples from 697 prostate cancer patients who underwent robotic-assisted laparoscopic radical prostatectomy at Taichung Veterans General Hospital (TVGH; Taichung, Taiwan) between 2012 and 2018. All subjects gave written informed consent prior to venous blood collection, and the investigation procedure was approved by the TVGH Institutional Review Board. Medical data collected at the time of diagnosis included prostate-specific antigen (PSA) levels, pathologic Gleason grades, clinical and pathologic T (tumor) and N (node) staging, cancer invasion sites (seminal vesicle, perineural, and lymphovascular regions) 29, D'Amico classification, and the biochemical recurrence (BCR) status 30.

### Selection and genotyping of SNPs

Based on other publications 31, 32, the *NUCB2* SNPs rs1330, rs214101, rs757081, and rs10766383 were chosen. Every SNP exhibited a minor allele frequency higher than 5%. 3 mL peripheral blood samples were used to extract genomic DNA using QIAamp DNA Blood Kits (Qiagen, CA, USA). Allelic discrimination was applied to the SNPs using previously established evaluation methods 33. The procedures we previously described 34, 35 were followed for RT-qPCR tests and RNA isolation.

### Analysis of clinical dataset

The GTEx portal (gtexportal.org/home/) is a comprehensive public resource for examining tissue-specific gene level and modulation. It provides open-access gene expression data, histology pictures, and quantitative trait loci (QTLs) 36.

### Statistical analysis

The Mann-Whitney U test and Fisher's exact test were used to evaluate the differences between the prostate cancer and control groups; *p*-values less than 0.05 were deemed statistically significant. Odds ratios (ORs) and their 95% confidence intervals (CIs) for the relationships between genotype frequencies and prostate cancer risk were computed using logistic regression. The Statistical Analytic System (SAS) software, version 9.1, was used to evaluate the gathered data.

## Results

The demographic and clinical features of 697 male Taiwanese patients with prostate cancer and 806 cancer-free healthy controls are distributed in Table 1. The prostate cancer group had a significantly larger proportion of patients over 65 (57.8%, n = 403) compared to the control group (11.7%, n = 94) (*p* < 0.001). Patients were more likely to exhibit pathologic Gleason scores of 1+2 (60.0%, n = 418), clinical T1 or T2 stages (86.2%, n = 601), pathologic T2 stage (52.9%, n = 369), and pathologic N0 stage (91.4%, n = 637). Patients presented with no seminal vesicle invasion (78.8%, n = 549) and no lymphovascular invasion (84.1%, n = 586). Furthermore, patients were classified as high-risk according to the D'Amico risk classification (66.2%, n = 352) and as having a low risk of BCR (68.6%, n = 478).

Table 2 exhibits the genotyping data for the *NUCB2* SNPs in health controls and patients with prostate cancer. The predominant genotypes were homozygous C/C for rs1330 and rs757081, homozygous G/G for rs214101, and homozygous T/T for rs10766383. None of the genotypes for the four *NUCB2* SNPs across the comparison groups showed a strong connection with prostate cancer after controlling for age (Table 2).

The distributions of clinical features and *NUCB2* genotypes within patients with prostate cancer were then compared. SNPs rs1330, rs214101 and rs757081 did not significantly affect clinicopathologic characteristics in patients with prostate cancer (Table 3&4). However, patients carrying at least one polymorphic G allele at rs10766383 (T/G + G/G genotypes) showed a lower risk of progression to clinical T3 or T4 stages (OR = 0.623; 95% CI: 0.394-0.983; *p* < 0.05), pathologic T3 or T4 stage (OR = 0.683; 95% CI: 0.488-0.956; *p* < 0.05), and perineural invasion (OR = 0.553; 95% CI: 0.365-0.836; *p* < 0.05) compared with the T/T genotype (Table 4). Importantly, the TG or GG genotypes at rs10766383 were associated with a reduced risk of clinical T3 + T4 stages (OR = 0.446; 95% CI: 0.234-0.849; *p* < 0.05) and perineural invasion (OR = 0.129; 95% CI: 0.017~0.990; *p* < 0.05) (Table 5) in patients with BCR.

**Figure 1:**
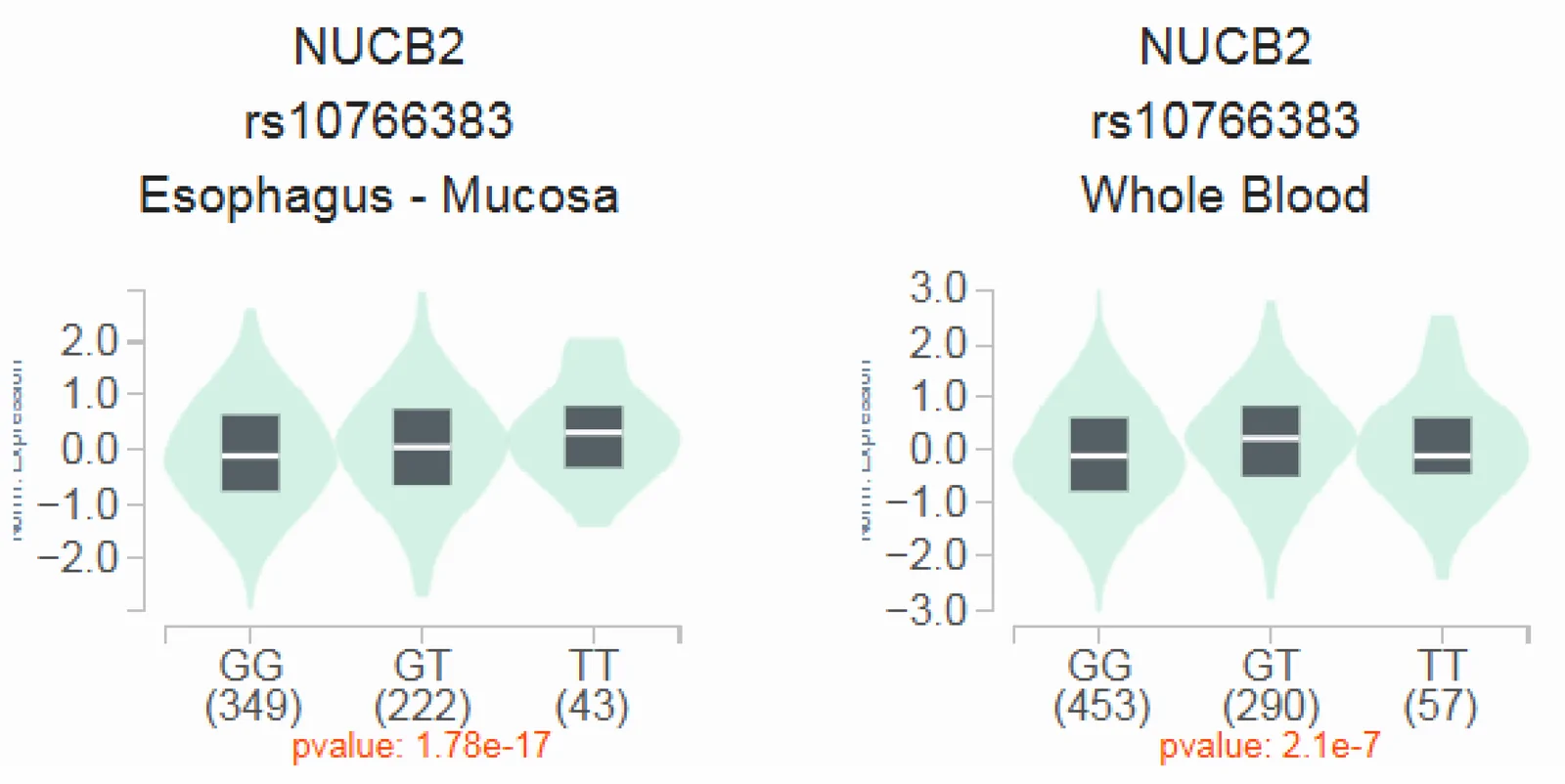
The *NUCB2* shows a substantial eQTL association with rs10766383 genotypes in mucosa and whole blood samples from the GTEx database.

The GTEx data showed that in mucosa and whole blood tissues, patients with the wild-type TT homozygous genotype had significantly higher levels of *NUCB2* than those with the GG allele of variant rs10766383 (*p* < 0.05; Figure 1).

## Discussion

The usefulness of biomarkers based on genetic abnormalities associated with malignancies in assessing risk, supporting prompt diagnosis, and forecasting treatment outcomes has been documented by different cancer studies 37, 38. Genetic polymorphisms, or individual differences in genomic sequences, are present in about 1% of the population. SNPs are the most common type of changes seen in repetitive sequences 39. The relevance of SNPs and other genetic alterations in defining and predicting pharmacotherapeutic actions in prostate cancer has lately been pointed out by a growing body of investigation 40. The significance of SNPs in tumor biology has also been highlighted by the systematic identification of functional variations associated with cancer risk, which has shown how SNPs in functional regions impact gene level and tumor susceptibility 39. *NUCB2* gene polymorphisms have been linked to oral cancer 33. However, the relation between *NUCB2* polymorphisms and prostate cancer still unknown. Therefore, we examined polymorphisms in the *NUCB2* gene and observed their differential distribution within prostate cancer patients. Our data indicated that the* NUCB2* SNP rs10766383 is linked with a declined risk of clinical T3+T4 stage, pathologic T3+T4 stage, and perineural invasion.

The development of prostate cancer is markedly associated by adipose tissue 41. Tumor cell proliferation, invasion, and metastasis are thought to be impacted by prostate cancer's direct or indirect interactions with adipocytes. Interestingly, the most common metastatic location in prostate cancer is bone 42, 43. Bone marrow adipocytes have been implicated in the formation and exacerbation of these bone metastases in a number of cases 44. For the past 20 years, numerous researchers have been examining the function of adipose tissue in the development of carcinogenesis and inflammation 45. Nesfatin-1 is essential for cell proliferation and the stimulation of cancer cell apoptosis, according to recent findings 25. Thyroid, breast, and colon cancer tissues had higher levels of nesfatin-1 than nearby non-cancerous tissues 46-48. Furthermore, in renal cell carcinoma, elevated nesfatin-1 expression was closely associated with advanced tumor stage and metastasis 49. In this work, we compared the genotypic and allelic distributions of *NUCB2* gene polymorphisms between patients with prostate cancer and health controls. The four *NUCB2* SNPs and total susceptibility to prostate cancer did not differ significantly. However, when we stratified prostate cancer patients according to clinical parameters, carriers of at least one G allele (T/G + G/G genotypes) at the *NUCB2* SNP rs10766383 were protected against developing clinical T3+T4 stage, pathologic T3+T4 stage, and perineural invasion. Importantly, GTEx data indicated that the wild-type TT homozygous genotype was linked with markedly higher *NUCB2* expression levels compared to the GG allele of variant rs10766383 variant in mucosa and whole blood tissues. Thus, *NUCB2* SNP rs10766383 may act as a negative factor of prostate cancer progression.

After radical prostatectomy or radiation therapy, BCR is a common clinical occurrence in PCA patients. An increasing PSA level, which suggests potential tumor recurrence or metastasis prior to the onset of clinical symptoms, is a common characteristic of BCR. In order to assess therapy results, forecast disease development, and direct future therapeutic approaches in PCA care, BCR monitoring is crucial. However, a rise in PSA levels following definitive treatment, such as surgery or radiation therapy, without any clinical or radiographic signs of illness is referred to as a prostate cancer BCR. It frequently shows recurrent or residual tumor activity and may precede the development of metastases, directing choices about salvage therapy or additional monitoring 9, 50, 51. In the current study, we also demonstrated that the TG or GG genotypes at rs10766383 were associated with a reduced risk of clinical T3 + T4 stages and perineural invasion compared with the TT genotype in patients with BCR. Therefore, the *NUCB2* SNP rs10766383 also plays a protective role in prostate cancer with BCR.

Finally, we should acknowledge the limitations of the current study. Although we found that the *NUCB2* SNP may play a protective role against prostate cancer progression, our study has several limitations. First, we lacked sufficient prostate cancer and normal control tissue samples for immunohistochemical staining. Therefore, further studies are needed to examine nesfatin-1 expression in prostate cancer patients and normal individuals to validate and support our SNP findings.

In summary, our investigation is the first to identify an association between *NUCB2* gene variants and prostate cancer. Compared with the wild-type T/T genotype, carriage of at least one G allele (T/G or G/G genotypes) at the *NUCB2* SNP rs10766383 was linked with a declined risk of clinical T3+T4 stage, pathologic T3+T4 stage, and perineural invasion. In addition, the TG/GG genotypes at rs10766383 were also linked to a lower risk of clinical T3+T4 stage and perineural invasion in patients with BCR. Our results suggest that *NUCB2* SNPs may play a critical role in prostate cancer progression and perineural invasion.

## Figures and Tables

**Table 1:** The distributions of demographical characteristics in 806 controls and 697 prostate cancers.

Variable	Controls (N = 806)	Patients (N = 697)	p value
**Age**			
≤ 65	712 (88.3 %)	294 (42.2 %)	P < 0.001*
> 65	94 (11.7 %)	403 (57.8 %)	
**Pathologic Gleason grade group**			
1+2		418 (60.0 %)	
3+4+5		279 (40.0 %)	
**Clinical T stage**			
1+2		601 (86.2 %)	
3+4		96 (13.8 %)	
**Pathologic T stage**			
2		369 (52.9 %)	
3+4		328 (47.1 %)	
**Pathologic N stage**			
N0		637 (91.4 %)	
N1		60 (8.6 %)	
**Seminal vesicle invasion**			
No		549 (78.8 %)	
Yes		148 (21.2 %)	
**Perineural invasion**			
No		185 (26.5%)	
Yes		512 (73.5 %)	
**Lymphovascular invasion**			
No		586 (84.1 %)	
Yes		111 (15.9 %)	
**D'Amico classification**			
Low risk/ Intermediate risk		345 (33.8 %)	
High risk		352 (66.2 %)	
**Biochemical recurrence**			
No		478 (68.6 %)	
Yes		219 (31.4 %)	

* p value < 0.05 as statistically significant.

**Table 2:** The adjusted odds ratio (AOR) and 95% confidence interval (CI) of prostate cancer associated with NUCB2/nesfatin-1 genotypic frequencies.

Variable	Controls (N = 806) (%)	Patients (N = 697) (%)	AOR (95% C.I.)	p value
**rs1330**				
CC	308 (38.2%)	251 (36.0%)	1.000 (reference)	
CT	381 (47.3%)	341 (48.9%)	1.003 (0.778~1.292)	P = 0.984
TT	117 (14.5%)	105 (15.1%)	0.992 (0.694~1.420)	P = 0.966
CT + TT	498 (61.8%)	446 (64.0%)	1.002 (0.887~1.128)	P = 0.998
**rs214101**				
GG	559 (69.4%)	460 (66.0%)	1.000 (reference)	
GA	211 (26.2%)	219 (31.4%)	1.177 (0.908~1.525)	P = 0.219
AA	36 (4.4%)	18 (2.6%)	0.886 (0.448~1.124)	P = 0.138
GA + AA	247 (30.6%)	237 (34.0%)	1.035 (0.914~1.173)	P = 0.586
**rs757081**				
CC	318 (39.5%)	261 (37.4%)	1.000 (reference)	
CG	379 (47.0%)	338 (48.5%)	1.032 (0.802~1.327)	P = 0.808
GG	109 (13.5%)	98 (14.1%)	0.961 (0.666~1.387)	P = 0.831
CG + GG	488 (60.5%)	436 (62.6%)	1.008 (0.894~1.136)	P = 0.899
**rs10766383**				
TT	210 (26.1%)	187 (26.8%)	1.000 (reference)	
TG	402 (49.9%)	354 (50.8%)	1.100 (0.831~1.457)	P = 0.505
GG	194 (24.0%)	156 (22.4%)	1.008 (0.723~1.404)	P = 0.964
TG + GG	596 (73.9%)	510 (73.2%)	1.034 (0.906~1.181)	P = 0.617

The adjusted odds ratios (AORs) with their 95% confidence intervals (CIs) were estimated by multiple logistic regression models after controlling for age.

**Table 3:** Odds ratios (ORs) and 95% confidence intervals (CIs) of the clinical status and *NUCB2/nesfatin-1* rs1330 and rs214101 genotypic frequencies in 697 patients with prostate cancer.

Variable	rs1330				rs214101			
	CC (*N* = 251)	CT+TT (*N* = 446)	OR (95% CI)	p value	GG (N = 460)	GA+AA (N = 237)	OR (95% CI)	p value
Pathologic Gleason grade group								
1+2	153 (61.0%)	265 (59.4%)	1.000	0.691	281 (61.1%)	137 (57.8%)	1.000	0.402
3+4+5	98 (39.0%)	181 (40.6%)	1.066 (0.777~1.463)		179 (38.9%)	100 (42.2%)	1.146 (0.833~1.576)	
Clinical T stage								
1+2	224 (89.2%)	377 (84.5%)	1.000	0.083	394 (85.7%)	207 (87.3%)	1.000	0.124
3+4	27 (10.8%)	69 (15.5%)	1.518 (0.945~2.441)		66 (14.3%)	30 (12.7%)	0.865 (0.544~1.375)	
Pathologic T stage								
2	141 (56.2%)	228 (51.1%)	1.000	0.199	248 (53.9%)	121 (51.1%)	1.000	0.474
3+4	110 (43.8%)	218 (48.9%)	1.226 (0.898~1.672)		212 (46.1%)	116 (48.9%)	1.121 (0.819~1.535)	
Pathologic N stage								
N0	231 (92.0%)	406 (91.0%)	1.000	0.651	423 (92.0%)	214 (90.3%)	1.000	0.459
N1	20 (8.0%)	40 (9.0%)	1.138 (0.650~1.993)		37 (8.0%)	23 (9.7%)	1.229 (0.712~2.121)	
Seminal vesicle invasion								
No	203 (80.9%)	346 (77.6%)	1.000	0.307	365 (79.3%)	184 (77.6%)	1.000	0.601
Yes	48 (19.1%)	100 (22.4%)	1.222 (0.831~1.797)		95 (20.7%)	53 (22.4%)	1.107 (0.757~1.618)	
Perineural invasion								
No	70 (27.9%)	115 (25.8%)	1.000	0.546	125 (27.2%)	60 (25.3%)	1.000	0.599
Yes	181 (72.1%)	331 (74.2%)	1.113 (0.786~1.576)		335 (72.8%)	177 (74.7%)	1.101 (0.770~1.574)	
Lymphovascular invasion								
No	216 (86.1%)	370 (83.0%)	1.000	0.284	391 (85.0%)	195 (82.3%)	1.000	0.352
Yes	35 (13.9%)	76 (17.0%)	1.268 (0.821~1.957)		69 (15.0%)	42 (17.7%)	1.221 (0.802~1.858)	
D'Amico classification								
Low risk/ Intermediate risk	133 (53.0%)	212 (47.5%)	1.000	0.167	229 (49.8%)	116 (48.9%)	1.000	0.834
High risk	118 (47.0%)	234 (52.5%)	1.244 (0.913~1.696)		231 (50.2%)	121 (51.1%)	1.034 (0.756~1.415)	
Biochemical recurrence								
No	182 (72.5%)	296 (66.4%)	1.000	0.094	311 (67.6%)	167 (70.5%)	1.000	0.442
Yes	69 (27.5%)	150 (33.6%)	1.337 (0.952~1.877)		149 (32.4%)	70 (29.5%)	0.875 (0.622~1.230)	

ORs with their 95% CIs were estimated by logistic regression models.

**Table 4:** Odds ratios (ORs) and 95% confidence intervals (CIs) of the clinical status and *NUCB2/nesfatin-1* rs757081 and rs10766383 genotypic frequencies in 697 patients with prostate cancer.

Variable	rs757081				rs10766383			
	CC (*N* = 261)	CG+GG (*N* = 436)	OR (95% CI)	p value	TT (N = 187)	TG+GG (N = 510)	OR (95% CI)	p value
Pathologic Gleason grade group								
1+2	156 (59.8%)	262 (60.1%)	1.000	0.933	110 (58.8%)	308 (60.4%)	1.000	0.708
3+4+5	105 (40.2%)	174 (39.9%)	0.987 (0.722~1.349)		77 (41.2%)	202 (39.6%)	0.937 (0.666~1.318)	
Clinical T stage								
1+2	229 (87.7%)	372 (85.3%)	1.000	0.370	153 (81.8%)	448 (87.8%)	1.000	0.041*
3+4	32 (12.3%)	64 (14.7%)	1.231 (0.781~1.941)		34 (18.2%)	62 (12.2%)	0.623 (0.394~0.983)	
Pathologic T stage								
2	146 (55.9%)	223 (51.1%)	1.000	0.220	86 (46.0%)	283 (55.5%)	1.000	0.026*
3+4	115 (44.1%)	213 (48.9%)	1.213 (0.891~1.650)		101 (54.0%)	227 (44.5%)	0.683 (0.488~0.956)	
Pathologic N stage								
N0	238 (91.2%)	399 (91.5%)	1.000	0.882	170 (90.9%)	467 (91.6%)	1.000	0.783
N1	23 (8.8%)	37 (8.5%)	0.960 (0.557~1.654)		17 (9.1%)	43 (8.4%)	0.921 (0.511~1.658)	
Seminal vesicle invasion								
No	212 (81.2%)	337 (77.3%)	1.000	0.219	141 (75.4%)	408 (80.0%)	1.000	0.188
Yes	49 (18.8%)	99 (22.7%)	1.271 (0.867~1.864)		46 (24.6%)	102 (20.0%)	0.766 (0.515~1.140)	
Perineural invasion								
No	70 (26.8%)	115 (26.4%)	1.000	0.898	35 (18.7%)	150 (29.4%)	1.000	0.005*
Yes	191 (73.2%)	321 (73.6%)	1.023 (0.723~1.447)		152 (81.3%)	360 (70.6%)	0.553 (0.365~0.836)	
Lymphovascular invasion								
No	228 (87.4%)	358 (82.1%)	1.000	0.067	149 (79.7%)	437 (85.7%)	1.000	0.055
Yes	33 (12.6%)	78 (17.9%)	1.505 (0.970~2.336)		38 (20.3%)	73 (14.3%)	0.655 (0.424~1.011)	
D'Amico classification								
Low risk/Intermediate risk	137 (52.5%)	208 (47.7%)	1.000	0.221	88 (47.1%)	257 (50.4%)	1.000	0.435
High risk	124 (47.5%)	228 (52.3%)	1.211 (0.891~1.646)		99 (52.9%)	253 (49.6%)	0.875 (0.626~1.224)	
Biochemical recurrence								
No	187 (71.6%)	291 (66.7%)	1.000	0.177	123 (65.8%)	355 (69.6%)	1.000	0.483
Yes	74 (28.4%)	145 (33.3%)	1.259 (0.901~1.760)		64 (34.2%)	155 (30.4%)	0.839 (0.588~1.198)	

ORs with their 95% CIs were estimated by logistic regression models. * p < 0.05 as statistically significant.

**Table 5:** Odds ratios (ORs) and 95% confidence intervals (CIs) of the clinical status and *NUCB2/nesfatin-1* rs10766383 genotypic frequencies in 697 patients with prostate cancer with biochemical recurrence.

Variable	No biochemical recurrence (N=478)				Biochemical recurrence (N = 219)			
	TT (N = 123)	TG+GG (N = 355)	OR (95% CI)	p value	TT (N = 64)	TG+GG (N = 155)	OR (95% CI)	p value
Pathologic Gleason grade group								
1+2	87 (70.7%)	262 (73.8%)	1.000	0.508	23 (35.9%)	46 (29.7%)	1.000	0.364
3+4+5	36 (29.3%)	93 (26.2%)	0.858 (0.544~1.352)		41 (64.1%)	109 (70.3%)	1.329 (0.718~2.461)	
Clinical T stage								
1+2	112 (91.1%)	324 (91.3%)	1.000	0.943	41 (64.1%)	124 (80.0%)	1.000	0.013*
3+4	11 (8.9%)	31 (8.7%)	0.974 (0.474~2.003)		23 (35.9%)	31 (20.0%)	0.446 (0.234~0.849)	
Pathologic T stage								
2	75 (61.0%)	242 (68.2%)	1.000	0.146	11 (17.2%)	41 (26.5%)	1.000	0.143
3+4	48 (39.0%)	113 (31.8%)	0.730 (0.477~1.117)		53 (82.8%)	114 (73.5%)	0.577 (0.275~1.211)	
Pathologic N stage								
N0	122 (99.2%)	344 (96.9%)	1.000	0.163	48 (75.0%)	123 (79.4%)	1.000	0.479
N1	1 (0.8%)	11 (3.1%)	3.901 (0.498~30.532)		16 (25.0%)	32 (20.6%)	0.780 (0.393~1.551)	
Seminal vesicle invasion								
No	110 (89.4%)	323 (91.0%)	1.000	0.611	31 (48.4%)	85 (54.8%)	1.000	0.388
Yes	13 (10.6%)	32 (9.0%)	0.838 (0.425~1.655)		33 (51.6%)	70 (45.2%)	0.774 (0.432~1.386)	
Perineural invasion								
No	34 (27.6%)	133 (37.5%)	1.000	0.063	1 (1.6%)	17 (11.0%)	1.000	0.021*
Yes	89 (72.4%)	222 (62.5%)	0.638 (0.407~1.000)		63 (98.4%)	138 (89.0%)	0.129 (0.017~0.990)	
Lymphovascular invasion								
No	111 (90.2%)	333 (93.8%)	1.000	0.186	38 (59.4%)	104 (67.1%)	1.000	0.276
Yes	12 (9.8%)	22 (6.2%)	0.611 (0.293~1.275)		26 (40.6%)	51 (32.9%)	0.717 (0.393~1.307)	
D'Amico classification								
Low risk/Intermediate risk	71 (57.7%)	200 (56.3%)	1.000	0.789	17 (26.6%)	57 (36.8%)	1.000	0.146
High risk	52 (42.3%)	155 (43.7%)	1.058 (0.699~1.602)		47 (73.4%)	98 (63.2%)	0.622 (0.327~1.184)	

ORs with their 95% CIs were estimated by logistic regression models. * p < 0.05 as statistically significant.
